# Spontaneous Coronary Recanalization After Iatrogenic Type F Dissection: A Chronic Total Occlusion-Like Lesion Requiring Advanced Percutaneous Intervention

**DOI:** 10.7759/cureus.117089

**Published:** 2026-09-28

**Authors:** Joseph Maldonado-Suarez, Héctor Martínez González, Jomar N Machuca-Santiago, David R Bermúdez Núñez, Juan F Rodríguez Acosta

**Affiliations:** 1 Cardiology, Mayagüez Medical Center, Mayagüez, PRI

**Keywords:** acute coronary syndrome (acs), arterial dissection, chronic total occlusion (cto), complex percutaneous coronary intervention (pci), coronary stents, intravascular ultrasound (ivus), spontaneous recanalization

## Abstract

Spontaneous coronary recanalization after a Type F dissection may behave as a chronic total occlusion (CTO)-like lesion despite angiographic flow, requiring CTO-level expertise to ensure true lumen wiring and optimal stent deployment. We present the case of a 70-year-old woman who developed spontaneous coronary recanalization after an attempted percutaneous coronary intervention complicated by a Type F dissection, with the recanalized right coronary artery (RCA) developing a complex CTO-like recanalized segment despite restoration of antegrade flow. After treatment with contemporary CTO strategies using intravascular ultrasound (IVUS) guidance, symptoms resolved. This case highlights the importance of recognizing the unique morphology and physiology of recanalized vessels and underscores the role of advanced imaging in the evaluation of complex post-dissection coronary anatomy.

## Introduction

Coronary artery dissection remains one of the most feared  complications of percutaneous coronary intervention (PCI), particularly Type F  dissections, defined as abrupt vessel closures with absent antegrade perfusion,  rendering thrombolysis in myocardial infarction (TIMI) 1 or TIMI 0 flow [1,2]. Although uncommon, these events carry significant morbidity, including myocardial infarction, hemodynamic  instability, and the potential need for emergency revascularization. When  true-lumen entry cannot be achieved, patients are often managed conservatively,  yet the long-term behavior of these lesions is variable and not fully  understood [1,3].

One rare but increasingly recognized phenomenon following  acute vessel occlusion is spontaneous coronary recanalization, in which the  previously obstructed artery reopens without further intervention. Historically  identified in autopsy studies, spontaneous recanalization has since been  characterized angiographically by irregular, braided, or pseudodissection-like  luminal patterns [3,4]. Advanced  intracoronary imaging modalities such as intravascular ultrasound (IVUS) and  optical coherence tomography (OCT) have demonstrated that these angiographic  findings correspond to multiple microchannels separated by smooth septa,  producing the classic "lotus root", "Swiss cheese", "honeycomb", and  "spider web" appearance [4,5].  Although the mechanisms underlying microchannel formation remain incompletely  defined, proposed etiologies include recanalization of organized thrombus,  healing of spontaneous or procedure-related dissection, and embolic events [3,4].

Clinically, spontaneous recanalization presents a diagnostic  and therapeutic challenge. Angiographic severity often underestimates the  functional significance of these lesions, and symptoms may persist despite the  appearance of partial flow restoration. The recanalized segment may behave  similarly to a chronic total occlusion (CTO)-like lesion, with complex dissection  planes and tortuous microchannels that remain as flow-limiting architecture and  complicate guidewire passage, requiring specialized CTO techniques for definitive revascularization [5-7].

We present a case of spontaneous coronary recanalization  following an attempted PCI, complicated by a Type F dissection, in which the  recanalized right coronary artery (RCA) evolved into a CTO-like lesion. The lesion was successfully  treated using contemporary CTO strategies with IVUS guidance, resulting in  complete symptom resolution. This case illustrates that spontaneous  recanalization may be angiographically reassuring yet physiologically  obstructive, prompting revascularization with pre-emptive intravascular imaging  and a CTO-style strategy when symptoms persist. The physiological significance of residual disease may vary and requires appropriate clinical or physiological assessment when indicated.

## Case presentation

A 70-year-old woman with a medical history of arterial hypertension, hyperlipidemia, and diabetes mellitus presented to our clinic for a second opinion due to persistent exertional angina following a complicated elective PCI performed at an outside hospital. Four weeks earlier, she had undergone an elective PCI of the RCA for obstructive coronary disease. During that procedure, an iatrogenic Type F dissection occurred, resulting in acute vessel closure. Multiple attempts to re-enter the true lumen were unsuccessful, and the procedure was aborted. During the attempted rescue of the acute vessel closure, the patient developed an acute myocardial infarction requiring admission to the intensive care unit. Her hospitalization was further complicated by a large right femoral hematoma requiring blood transfusion and prolonged monitoring.

At the time of evaluation in our clinic, the patient continued to experience a Canadian Cardiovascular Society (CCS) class III angina despite optimal medical therapy. Review of the angiographic compact disc images from the index procedure demonstrated complete occlusion of the RCA with extensive dissection planes and intramural hematoma (Videos 1-2). Given her persistent symptoms, a repeat coronary angiography was recommended.

**Video 1:** Diagnostic coronary angiography of the right coronary artery showing proximal diffuse disease

**Video 2:** Coronary angiography showing an acute Type F dissection of the right coronary artery

Ten weeks after the initial event, repeat catheterization revealed spontaneous partial recanalization of the RCA. Angiography demonstrated a severely narrowed, irregular lumen with multiple channels consistent with a CTO formed within healed dissection planes (Video 3). Despite the presence of antegrade flow, the vessel exhibited severe stenosis and complex architecture impeding laminar flow, and the patient continued to experience CCS class III angina. 

**Video 3:** Coronary angiography 10 weeks after the first intervention showing spontaneous coronary recanalization of the right coronary artery

Given the severe residual obstruction and complex multilumen architecture of the recanalized RCA, an antegrade CTO-style wiring strategy was undertaken. Right femoral access was obtained using ultrasound guidance and a 7 Fr JR4 guiding catheter. A Runthrough NS Extra Floppy wire (Terumo, Somerset, New Jersey, United States) was initially advanced, followed sequentially by a Pilot 200 (Abbott, Abbott Park, Illinois, United States) and Judo-6 wire (Boston Scientific, Marlborough, Massachusetts, United States), with a Mamba microcatheter (Boston Scientific, Marlborough, Massachusetts, United States) used for support and wire escalation (Figure 1).

**Figure 1:**
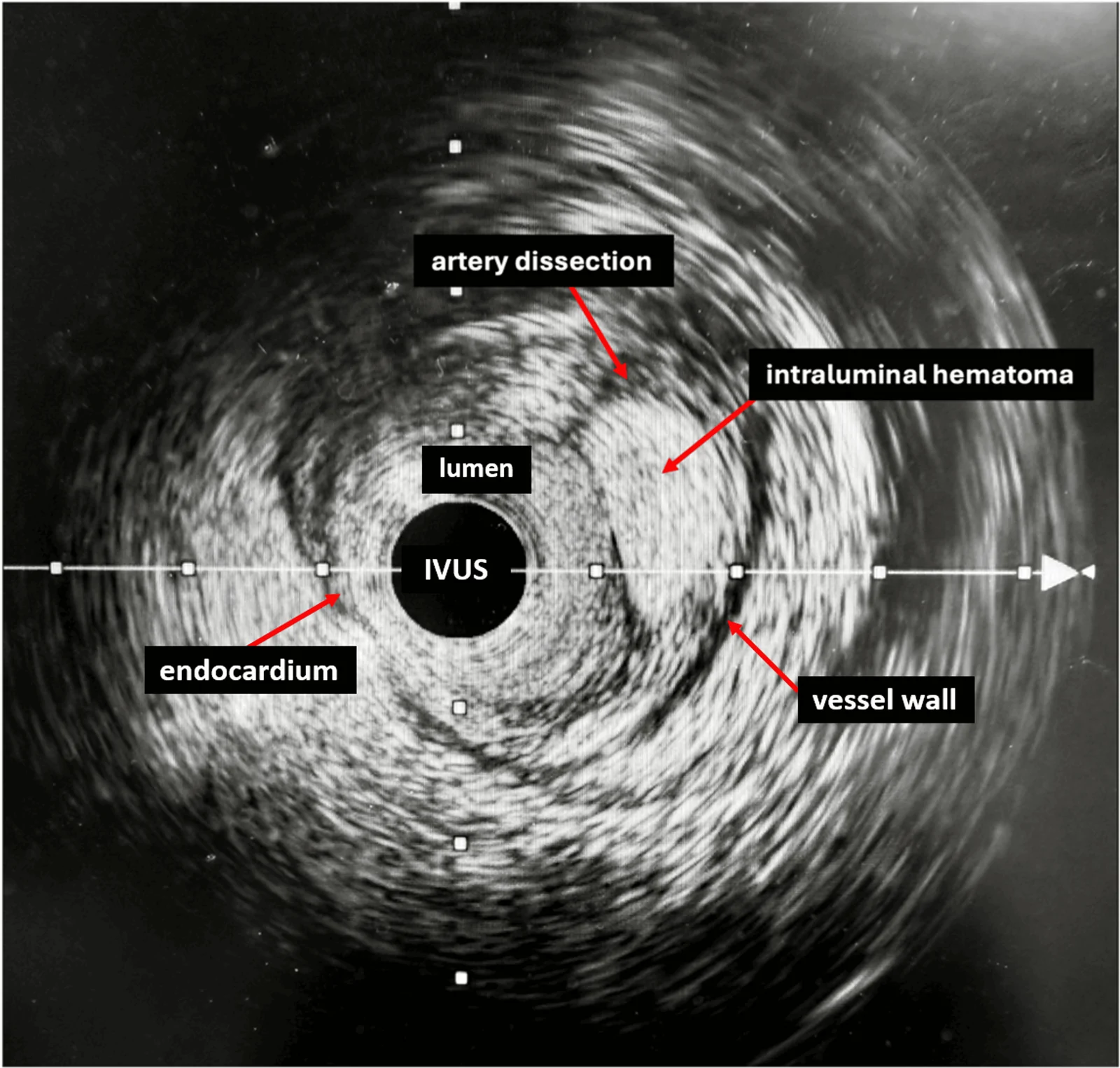
IVUS showing the dissection of the right coronary artery along the hematoma IVUS: intravascular ultrasound

A Fighter Plus wire (Boston Scientific, Marlborough, Massachusetts, United States) ultimately crossed the lesion. IVUS was then performed to assess the wire position and vessel architecture and demonstrated the wire within the distal true lumen. The previously dissected segment demonstrated a 68 mm lesion with severe residual luminal narrowing and multiple irregular channels consistent with complex dissection-related architecture. The proximal and distal reference vessel diameters were approximately 3.5 mm and 3 mm, respectively. Despite restoration of antegrade flow, the lesion remained severely obstructive angiographically. Given the patient's persistent CCS class III angina despite optimal medical therapy and the severe residual angiographic obstruction, revascularization using a CTO-style strategy was undertaken. No objective physiological or ischemia testing was available at that time to independently document ischemia. This confirmation established the appropriate intraluminal course before lesion preparation and stent implantation. Following true-lumen confirmation, the lesion was predilated with a 2×15 mm balloon at 12 atm, followed by the deployment of three Synergy Megatron drug-eluting stents (3.5×8 mm, 3×32 mm, and 3×28 mm) (Boston Scientific, Marlborough, Massachusetts, United States) from proximal to mid RCA. Post-dilation was performed with a 3.5 mm noncompliant balloon at 16 atm. Final IVUS demonstrated excellent stent expansion and apposition, with a minimal stent area of 8.2 mm² and no residual dissection (Figure 2). Final angiography demonstrated TIMI 3 flow (Video 4).

**Figure 2:**
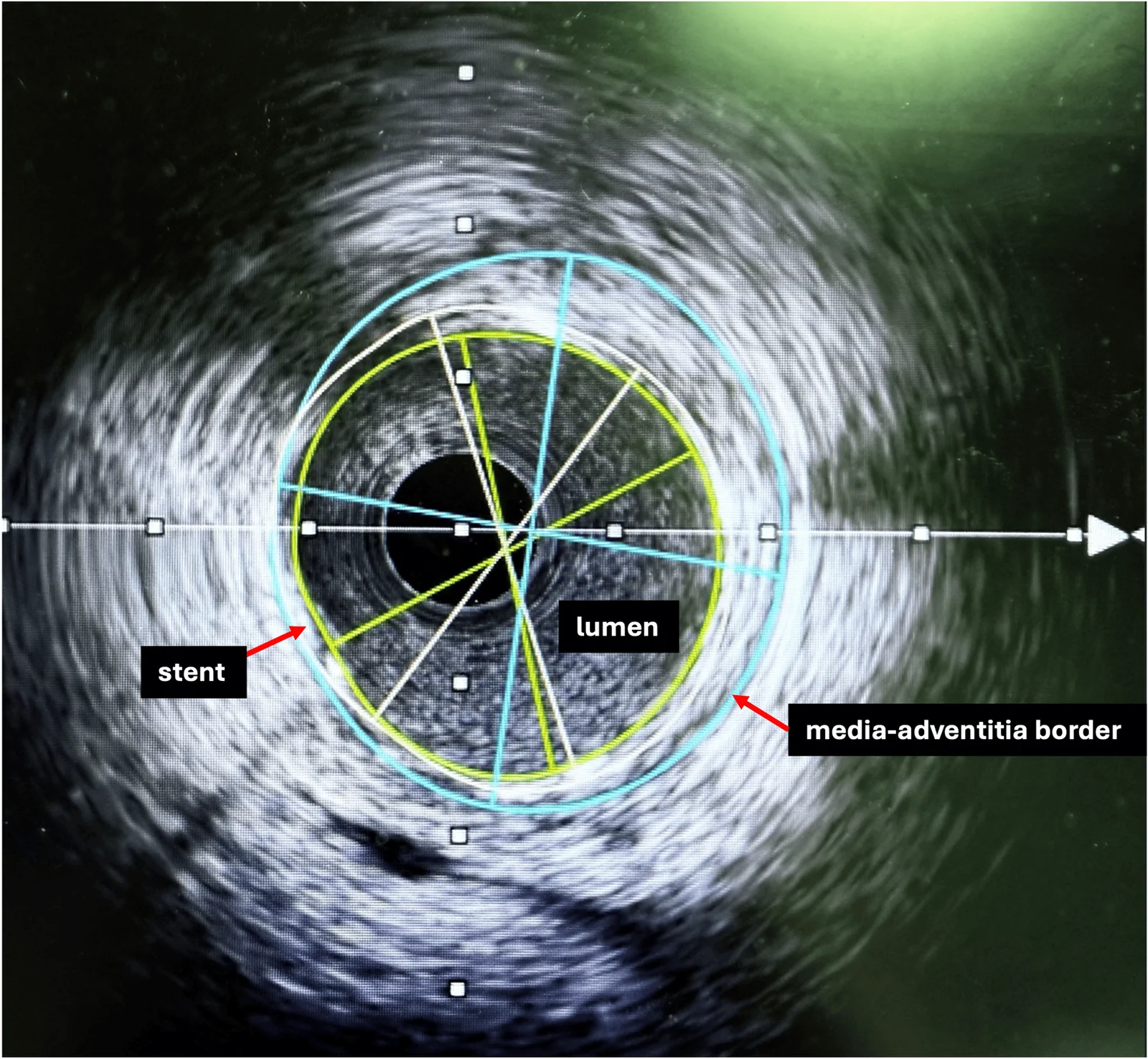
Intravascular ultrasound of the right coronary artery status post-percutaneous coronary intervention showing excellent stent expansion and apposition The lumen area is encircled in green, and the media-adventitia border is demarcated by the blue circle. The stent is demarcated by the white circle.

**Video 4:** Right coronary artery status post-percutaneous coronary intervention

The patient was discharged the same day on dual antiplatelet therapy and aggressive secondary prevention with aspirin and ticagrelor. At the one- and three-month follow-ups, she reported complete resolution of angina and demonstrated a favorable clinical recovery. Longer-term follow-up will be important given the extensive coronary dissection and unusual underlying vascular biology. The case timeline is summarized in Table 1. 

**Table 1:** Case timeline CCS: Canadian Cardiovascular Society; CTO: chronic total occlusion; ICU: intensive care unit; IVUS: intravascular ultrasound; OMT: optimal medical therapy; PCI: percutaneous coronary intervention; RCA: right coronary artery; TIMI: thrombolysis in myocardial infarction

Case timeline	
Pre-procedure	An elderly woman with cardiovascular risk factors underwent elective PCI of the RCA at an outside hospital. The procedure was complicated by a Type F dissection, resulting in abrupt vessel closure and unsuccessful attempts at true‑lumen entry, leading to an acute myocardial infarction requiring ICU admission
Post-procedure	Despite optimal medical therapy, she continued to experience CCS class III angina. Ten weeks after the index PCI, repeat coronary angiography demonstrated spontaneous recanalization of the RCA with persistent dissection planes, creating a CTO-like lesion despite the presence of antegrade flow
Diagnosis and management	Given persistent symptoms, she underwent complex PCI using an antegrade wiring strategy with microcatheter support. An antegrade wiring strategy with sequential wire escalation supported by a microcatheter to navigate the dissected architecture and IVUS confirmed the true‑lumen positioning. Three Synergy Megatron drug‑eluting stents were deployed with excellent expansion and apposition. Final angiography showed restored TIMI 3 flow without complications
Outcome and clinical pearl	The patient was discharged the same day with complete resolution of angina. At the one- and three-month clinical follow-ups, she remained free of anginal symptoms and had experienced no intervening cardiovascular events. This case illustrates that spontaneous recanalization of an extensively dissected coronary artery may result in a CTO-like lesion despite angiographic antegrade flow. In selected symptomatic patients, intravascular imaging and CTO-oriented wire techniques may facilitate the successful treatment of complex post-dissection coronary anatomy. The findings are consistent with but do not establish the proposed mechanism of spontaneous recanalization and subsequent vessel wall remodeling

## Discussion

Procedure-related coronary artery dissections are classified using the National Heart, Lung, and Blood Institute (NHLBI) system, which ranges from Type A to Type F based on angiographic appearance and hemodynamic impact [1,2]. Types A-E represent increasing severity of luminal disruption, contrast staining, and propagation,  whereas Type F dissections are characterized by abrupt vessel closure with  absent antegrade perfusion (TIMI 0-1 flow) and represent the most severe form.  These events are among the most serious complications of PCI because of their association with acute myocardial infarction,  hemodynamic instability, and the potential need for emergent  revascularization [2].

In this case, the patient experienced a procedure-related Type F dissection during elective PCI of the RCA, resulting in complete vessel occlusion and unsuccessful attempts at true-lumen entry. When such dissections cannot be successfully rewired or sealed, conservative management may be considered in selected patients, although the subsequent evolution of these lesions is variable. One uncommon but clinically important outcome is spontaneous coronary recanalization, in which a previously occluded artery develops renewed antegrade flow without further intervention [3].

Historically described in autopsy studies, spontaneous recanalization has subsequently been recognized angiographically by irregular, braided, or pseudodissection-like luminal patterns. Intravascular imaging with IVUS and OCT has demonstrated that some of these appearances correspond to multiple microchannels separated by smooth septa, producing characteristic "lotus root", "Swiss cheese", "honeycomb", or "spider web" morphologies [4,5]. These microchannels may represent organized pathways through previously thrombosed or dissected segments and may arise through mechanisms including thrombus organization and recanalization, healing of spontaneous or procedure-related dissection, or organization of embolic thrombus [3,4].

Although angiography may demonstrate restoration of antegrade flow, angiographic recanalization alone does not establish normalization of coronary physiology. Prior reports have described abnormal physiological or ischemia assessments in selected recanalized lesions [6]; however, the available literature is limited and does not establish that spontaneously recanalized Type F dissections are generally hemodynamically significant. In the present case, the recanalized RCA demonstrated antegrade flow but retained complex dissection planes and a multilumen appearance. No formal physiological assessment with fractional flow reserve (FFR) or instantaneous wave-free ratio (iFR), or dedicated ischemia testing, was performed. Therefore, the characterization of the lesion as a CTO-like lesion reflects its complex anatomical and procedural characteristics rather than an objectively measured physiological definition.

The evidence base for treating spontaneously recanalized Type F dissections specifically as CTO-like lesions is limited. There are no established comparative data defining CTO PCI as the preferred strategy for this particular lesion subset. The application of contemporary CTO techniques in this setting is therefore largely extrapolated from the broader CTO literature. The presence of multiple luminal channels, dissection planes, and potentially organized thrombotic material can create technical challenges analogous to those encountered during CTO intervention [4,7]. In this case, antegrade wiring with microcatheter support and IVUS facilitated identification of the true lumen, wire advancement, and subsequent stent optimization. These observations demonstrate the technical applicability of such techniques in a selected case but do not establish their superiority over alternative strategies.

The use of intravascular imaging may be particularly useful in complex post-dissection anatomy because IVUS can help define true- and false-lumen relationships, guidewire position, vessel wall boundaries, and stent expansion and apposition [5]. In this patient, IVUS was instrumental in confirming true-lumen positioning and optimizing the final stent result. The use of a high-radial-strength drug-eluting stent was selected based on the proximal RCA anatomy, vessel caliber, and the need for adequate scaffolding across the previously dissected segment. However, the favorable procedural and clinical outcome in this individual patient should not be interpreted as evidence establishing the efficacy or safety of this approach.

Published reports have described a range of clinical presentations following spontaneous coronary recanalization, including persistent symptoms in some patients [3-6]. However, because the available evidence consists predominantly of case reports and small observational experiences, the frequency with which patients remain symptomatic or require subsequent intervention cannot be reliably determined. In the present case, persistent CCS class III angina despite medical therapy provided the clinical rationale for further evaluation and revascularization.

This case therefore illustrates a potential management scenario rather than a general treatment paradigm. In a symptomatic patient with spontaneous recanalization following an extensive Type F dissection, persistent complex luminal architecture may present substantial technical challenges despite the presence of antegrade angiographic flow. Intravascular imaging and CTO-oriented wiring techniques may assist procedural planning and guidewire navigation in selected cases. However, angiographic appearance alone should not be equated with physiological significance, and decisions regarding revascularization should incorporate the patient's symptoms, objective evidence of ischemia when available, anatomical characteristics, and clinical context.

In this selected patient, IVUS-guided antegrade PCI using CTO-oriented techniques was technically feasible and was associated with a favorable clinical outcome. However, this single-case observation does not establish the safety or efficacy of this approach for spontaneously recanalized coronary dissections in general.

## Conclusions

Spontaneous recanalization after iatrogenic Type F coronary dissection may result in complex CTO-like anatomy despite restoration of antegrade flow. In this symptomatic patient, IVUS-guided antegrade PCI with microcatheter support enabled successful revascularization. This single-case observation demonstrates technical feasibility but does not establish the efficacy, safety, or optimal management strategy for similar lesions.
